# Neuropsychological assessment of attention in children with spina bifida

**DOI:** 10.1186/1743-8454-6-6

**Published:** 2009-05-28

**Authors:** Anja Vinck, Reinier Mullaart, Jan Rotteveel, Ben Maassen

**Affiliations:** 1 Department of Medical Psychology, Radboud University Nijmegen Medical Centre, PO Box 9101, 6500 HB Nijmegen, the Netherlands; 2 Department of Pediatric Neurology, Radboud University Nijmegen Medical Centre, PO Box 9101, 6500 HB Nijmegen, the Netherlands

## Abstract

**Background:**

Children with the severe form of spina bifida (SBM: spina bifida with myelomeningocele with accompanying hydrocephalus) may manifest attention deficits, and have a similar psychological profile to children with hydrocephalus due to other etiologies. It is unclear to what extent tests to assess attention in SBM are confounded by the accompanying cognitive or visual-motor impairments. The aim of this study was to analyse attention functions by administering two different types of attention tests, one with high and the other with low cognitive and motor requirements. This enabled the possible interaction between attention and cognitive and motor impairment to be assessed.

**Methods:**

The study group comprised 31 children with SBM with shunted hydrocephalus. Twenty children with SB-only formed a closely matched comparison group. Of these, 19 children with SBM and 18 with SB had a full-scale IQ (FSIQ) higher than 70. All had undergone spinal surgery and all children with SBM had been shunted within the first months of life. Between 6 and 15 years of age, the children were assessed on focused and sustained attention, encoding, and distractibility/impulsivity, using both traditional tests and computerized attention tests.

**Results:**

Compared to the SB group, attention scores of children with SBM were lower on the traditional tests, but when interfering cognitive and visual-motor requirements were eliminated using the computerised tasks, most differences disappeared. Furthermore, in contrast to traditional attention tasks, computerized tests showed no significant correlations with IQ-scores and visual-motor skills.

**Conclusion:**

Assessment of attention functions in children with SBM by traditional tests may be misleading, because this paediatric population with complex cerebral malformations has difficulty with the cognitive and visual-motor requirements. To control for these interactions, the use of both traditional and computerized attention tests is recommended.

## Background

Spina bifida (SB) is a neuro-embryological disorder with complex physical and neuropsychological morbidity. The majority of children with myelomeningocele (SBM), the severe form of SB, develop hydrocephalus and associated cerebral malformations of the posterior cortex and white matter, midbrain, cerebellum, and corpus callosum [1-4]. The intellectual skills of children with SBM are often in the low average to average range. The cognitive profile of children with SBM is in many respects similar to that of children with hydrocephalus of different etiologies, with verbal skills being typically more advanced than nonverbal problem-solving skills [5-8]. Cognitive impairments associated with hydrocephalus and/or SBM may affect academic performance [9], and include deficits in visual perception [10], motor skills [11-13], and memory [14,15]. The relatively intact verbal skills in children with SBM may be deficient in areas such as verbal memory [15], discourse and idiom comprehension [16], speech fluency, and articulation [17]. Thus, in these children the cerebral malformations have considerable impact on their neurobehavioral outcomes.

Recent attention studies indicate that children with SBM tend to have difficulties with encoding, sustaining, focusing, and shifting attention [18-20]. Both the parents and teachers of these children often report problems in these areas, as well as in closely related executive functions [19,21,22]. A drawback of current psychometric measures of attention is that performance on most attention tests depends heavily on additional cognitive and motor functions, which may cause measurement bias in cases where these additional functions are deficient, to such an extent that they interfere with execution of the primary attention task. Consequently, the assessment of attention in SBM might be confounded by deficits in the visual-motor [8], verbal memory [15], and fine motor domains [11,23,24]. A similar kind of reasoning was suggested by Fletcher *et al. *[19], who argued that the observed differences between children with and those without hydrocephalus on measures of focused attention, were primarily attributable to motor deficiencies rather than to attention deficits *per se*. In contrast, the findings by Brewer *et al. *[18] and Loss *et al. *[20] suggested that even when demands on response speed and motor control were minimized, children with hydrocephalus continued to display attention problems. These studies demonstrated that interpretation of test results on attention in this population was not as straightforward as in a population without visual-motor deficits. This led us to hypothesize that in order to assess attention in children with SBM adequately, special tests are required that would take into account the interference from other deficits and preferably eliminate any confounding factors.

The aim of the present study was to analyze to what extent in children with SBM and associated complex cerebral malformations, poor attention performance on psychometric assessments is due to interference by cognitive or visual-motor deficits. To control for such potential interference we used an extensive test battery comprising both traditional attention tests requiring more complex cognitive or visual-motor skills, as well as simple, computer-based tasks designed to minimize the potential effects of any additional deficits. In these latter tasks the type of attention function tested was maintained, although the response only required a single button press. The performances of children with SBM on both the complex and simple tasks were compared with a closely matched control group of children diagnosed with SB only, with no or minor cognitive and visual-motor deficits. The hypothesis tested was that the poorer performance in the children with SBM is not caused by an attention deficit, but by the concomitant cognitive and visual motor deficits. To further analyze the possible interfering effects of these deficits, we calculated correlations between measures of attention and overall intelligence levels, and conducted separate analyses with a subgroup of children with an IQ ≥ 70. Finally, we compared all performance results of the SBM group with a closely matched control group of children diagnosed with SB only.

## Methods

### Study participants

The experimental procedures of our study were approved by the Regional Committee on Research Involving Human Subjects and written consent was obtained from the parent(s) of all children. Seventy-eight children with SB born between January 1988 and December 1997 and referred for spinal surgery to the Radboud University Nijmegen Medical Centre, were invited to participate in the current study because their neonatal condition had been systematically assessed earlier by our research group in the context of a larger research project. Twenty-seven of the selected children could not be examined adequately for various reasons: refusal to participate, insufficient command of the Dutch language and profound retardation precluding formal testing. The final study group thus comprised 51 children of whom 31 had SBM and 20 SB. All had undergone spinal surgery and all children with SBM were shunted within the first months of life. The medical characteristics of the children are presented in Table 1. The main inclusion criterion was the presence of a congenital defect in the closure of one or more vertebral arches in combination with a median skin defect and/or a cystic or lipomatous lump on the back, and/or a developmental anomaly of the spinal cord confirmed by MRI (magnetic resonance imaging). Spina bifida was further specified according to the following three characteristics: (1) Type of spinal anomaly scored with diagnostic codes as closed (ICD-10 code Q76.0) or open (ICD-10 code Q05); (2) Cerebral comorbidity scored as hydrocephalus (ICD-10 code Q03) with the diagnosis being based on specified MRI or CT (computed tomography) features, and Arnold-Chiari II malformation (ICD-10 code Q07) or corpus callosum dysgenesis (ICD-10 code Q04.0) with diagnoses based on MRI analysis; (3) Neurological impairment of the lower part of the body scored as the uppermost affected spinal segment with decreased sensibility and/or decreased intentional movement [25]. Sensibility was defined as behavioural reactions on pin prick and light touch. Intentional movement was defined as non-stereotypical, non-reflex motion.

**Table 1:** Patient characteristics for the SB and SBM groups

	SB (n = 20)	SBM (n = 31)
Age at spinal surgery (weeks) (quartile range)	median = 56 24 – 116	median = 0 0 – 0
Type of defect	Open = 3 Closed = 17	Open = 31 Closed = 0
Arnold-Chiari II malformation confirmed by MRI	0^1^	26^2^
Callosal dysgenesis confirmed by MRI	0^1^	13^2^
Level of spinal impairment (paresis)	L4-S3 (mean = L5)	T10-S1 (mean = L3)

*Notes*:

^1 ^one unknown (no MRI performed); ^2 ^five unknown (no MRI performed)

SB = spina bifida only; SBM = spina bifida with myelomeningocele

To assess the effect of hydrocephalus and associated neuropathology, we formed two subgroups, one comprising SB children with myelomeningocele (SBM) and the second patients without myelomeningocele (SB). All children with SBM had an open spinal defect while most children with SB had a closed defect. Arnold-Chiari II malformation was confirmed by MRI for all children with SBM except for five, and 50% of these had callosal dysgenesis, with no such cases in the SB cohort. The total study group comprised 28 girls and 23 boys and was between 6 and 15 years of age at the time of testing. Mean ages and intelligence measures, based on the Wechsler-Intelligence Scale for Children, 3rd edition (WISC-III, Dutch version), [26], and the VMI [27], per subgroup are listed in Table 2. To control for the confounding effect of cognitive impairment, separate analyses were conducted after exclusion of children with a full-scale IQ (FSIQ) below 70. Table 2 also presents the IQ data of the resulting non-retarded subgroups (SB, n = 18, SBM, n = 19).

**Table 2:** Intelligence and visual-motor integration scores (mean, sd) for the complete SB and SBM groups and the non-retarded subgroups

		N	Age (years;months)	Age range	VIQ	PIQ	FSIQ	VMI
Complete group	SB	20	10;0	6;6 – 14;11	95.0 (15.4)	93.1 (16.4)	93.4 (16.1)	96.75 (9.78)
	SBM	31	10;5	6;4 – 15;1	80.2 (16.7)	67.7 (16.2)	71.9 (16.0)	80.94 (15.4)
Non-retarded group	IQ ≥ 70SB	18	9;7	6;6 – 14;11	97.8 (13.4)	96.1 (14.2)	96.5 (13.6)	98.33 (8.76)
	IQ ≥ 70SBM	19	9;9	6;4 – 12;9	90.7 (8.6)	76.8 (11.6)	82.7 (8.0)	89.47 (8.1)

*Notes*:

SB = spina bifida; SBM = spina bifida with myelomeningocele; VIQ = verbal intelligence quotient; PIQ = performance intelligence quotient; FSIQ = full-scale intelligence quotient [26];

VMI = Visual-Motor Integration score [27]

In contrast to the children with SB only, all children with SBM had significantly lower scores on performance IQ (PIQ) than on verbal IQ (VIQ; complete SBM subgroup: *F*(1,30) = 42.02, *p *< 0.001; non-retarded SBM subgroup:*F*(1,18) = 25.57, *p *< 0.001). As to the non-retarded children, the difference between SBM and SB was only significant for PIQ (*F*(1,35) = 20.38, *p *< 0.001), FSIQ (*F*(1,35) = 14.34, *p *< 0.001), and VMI (*F*(1,35) = 10.2, *p *< 0.005) and not for VIQ (*F*(1,35) = 3.74, *p *> 0.05).

### Testing procedures

All children underwent an extensive neuropsychological assessment as part of a larger study [28]. For the assessment of attention functions, two series of tests were administered (Additional file 1) measuring various dimensions of attention with the first series comprising traditional tests and subtests from the WISC-III [26]. These are considered more complex tasks in that their performance not only requires attention but also the recruitment of additional cognitive and motor functions, more specifically, visual-motor integration (drawing), or verbal reproduction. The arithmetical task is also classified as complex because of the special cognitive skill it gauges, which in itself is irrelevant for the assessment of attention *per se*.

In the series of 'simple' computerized attention tasks we presented, motor output was reduced to a button press and the tasks thus put minimal demands on visual-motor functions, verbal skills or targeted cognition, although it should be noted that the attentional demands of the computerized tasks were similar to those in the traditional tests. Attention is operationalized as efficient information processing as reflected by speed (reaction times) and accuracy (errors to stimuli, e.g. false alarms, misses). We opted for the computerized attention subtests from the Amsterdam Neuropsychological Tasks (ANT [29]) as their validity and sensitivity for children in the same age range has been previously demonstrated [30,31]. Especially, the measurement of reaction time in combination with accuracy contributes to the sensitivity and distinctive value of the ANT [32]. Participants are asked to place their index finger of each hand on the corresponding left and right button of a static computer mouse (fixed to the table top), and to press the right button in case of a 'Yes' or the left button in case of a 'No' answer. The dependent variables for all ANT tasks were mean reaction time (RT, taken to reflect speed of information processing), the number of errors (incorrect responses and omissions), and response variability as expressed by RT standard deviations (SD).

We assessed the following four dimensions of attention: focused attention (i.e. the ability to select relevant stimuli from a broad array), sustained attention (the capacity to maintain focus and alertness over time), encoding (the ability to hold information 'in mind' for immediate manipulation or action), and distractibility/impulsivity. For each domain, both the 'more complex' traditional and the 'simpler' computer tasks were administered.

Focused attention was assessed using the traditional Symbol Search and Coding subtests of the WISC-III intelligence test [26] whose scores are based on age norms allowing comparisons across age groups. In the ANT's Focused Attention task, a bowl containing different kinds of fruit is presented on the screen. The child is instructed to press the 'Yes' button if the cherries (the target fruit) are located at the upper or lower part of the screen and to press the 'No' button if there are no cherries in the bowl or when they are placed at the right- or left-hand side of the bowl. Note that this implies a two-step decision rule.

Sustained attention was gauged with the Bourdon-Vos Test [33], a cancellation test requiring high-speed visual selectivity and a repetitive motor response. Like most cancellation tasks, this test assesses many functions, but above all the capacity for focused and sustained attention [34] given its length and duration. Children are instructed to cross out the target items, i.e. the dot patterns with four dots on a sheet of paper covered in three- four-, and five-dot patterns. Dependent variables are mean time (in sec) taken to complete one row of 24 patterns with the standard deviation (sd) of row times indicating fluctuations in attention. In the Sustained Attention subtest of the ANT, which lasts between 7 and 10 minutes, a house with four windows is presented on the screen. In each trial one animal appears at a random window and the child is asked to press the 'Yes' button when the target animal (e.g. a mouse) appears in one of the windows. In all other cases the 'No' button needs to be pressed.

Encoding was measured using the WISC Digit Span and Arithmetical subtests and the ANT Memory Search test. Here, a house with four different animals behind its four windows is presented on the screen. The combination of animals differs per stimulus presentation. The child is asked to memorize a particular target animal (e.g. the butterfly) and then to indicate whether the target animal appears in the subsequent pictures by pressing the 'Yes' button if it does and the 'No' button if it does not.

Distractibility/impulsivity was assessed with the Stroop colour-word test [35], comprising ten rows of ten items in each of its three conditions. The child is first asked to read colour words, then to name the colour of simple colour patches, and finally, in the condition of interest, to name the colour of the ink in which colour words were printed, with the two always being in conflict. Normally, reading the words is pre-potent over naming the ink colour and task performance requires attention to be directed to the colour in which the words are printed while the pre-potent response needs to be inhibited. The dependent variable was the time (in sec) taken to perform each condition. An interference score was computed by subtracting the performance time of the second (colour naming) condition from that of the third (interference) condition. The computer test comprised a *GoNoGo task*. After the presentation of a fixation point (x sign), an open or closed square is presented and the child is instructed to press the 'Yes' button when the open square is shown. In case of a closed square, no response is required, implicating that the child has to suppress the habitual response and has to wait for the next stimulus to appear.

For the computer tasks, demonstration and practice trials with feedback were used to reduce the impact of between-subject differences in task comprehension and to improve the reliability of the performance measures.

### Statistical analyses

Statistical analyses were performed using SPSS 14.0 for Windows. Parametric testing was not feasible for all variables since Levene's test for homogeneity and Shapiro-Wilks' test of normality were statistically significant. Therefore, nonparametric Mann-Whitney *U *tests were used to address the differences between SB and SBM on the different attention domains. Dependent variable factor scores were calculated per attention domain for complex and simple tests separately. Furthermore, the results of the nonparametric Mann-Whitney *U *tests were used to calculate effect sizes (ES) for each subtest [36]. To investigate whether the attentional outcomes of both task types (traditional *versus *computer-based) correlated with cognitive impairment, nonparametric Spearman's rho correlation coefficients were calculated with VIQ, PIQ, FSIQ and VMI. To explicitly eliminate the influence of cognitive impairment, for each of the two groups, all analyses were also conducted on the non-retarded (IQ ≥ 70) subgroups.

Because for some of its subtests norm data for certain age groups were incomplete, we used the raw data on the computerized ANT tasks in our statistical analyses rather than standardized scores even though for those tests where this was possible, additional analyses using standardized scores yielded similar results.

## Results

### Attention differences in SBM and SB (whole groups)

(Additional file 1) summarizes the performance outcomes for the SBM and SB groups on each dimension of attention per task type. Sample sizes vary between tasks because not all children were able to complete all tasks. As to the traditional 'complex' tasks, the differences between the two groups were statistically significant with medium to large effect sizes for the Symbol Search and Coding tasks (focused attention), Digit Span and Arithmetic (encoding), and Stroop 3 (distractibility/impulsivity). Non-significant differences but medium effect sizes were found for the standard deviation of the Bourdon-Vos test in the sustained attention domain. A small effect size was found for Stroop Interference of the distractibility/impulsivity domain.

The 'simple' computerized tasks did not yield statistically significant group differences nor large effect sizes for reaction-time or error measures, except for moderate significance (p < 0.05) for the subtest Focused Attention of the ANT. This subtest showed also medium to small effect sizes, which was also found for sustained attention RT. For the tests mentioned, the performance of the SBM patients was always inferior to that of the SB patients.

### Attention differences between the non-retarded SBM and SB patients

To further control for the effect of overall cognitive impairment, comparative group analyses were conducted separately for the non-retarded patients only (IQ ≥ 70, Additional file 1). Symbol Search and Coding (focused attention) were the only 'complex' tasks demonstrating statistically significant differences between the SB and SBM subgroups. None of outcomes on the computer tasks generated statistically significant differences. Except for Symbol Search and Coding, all effect sizes were small or showed no differences.

### Correlations between attention outcomes and VIQ, PIQ, and FSIQ scores

To statistically control for the confounding effect of overall cognitive impairment, nonparametric Spearman's rho correlation coefficients were calculated between each task and VIQ, PIQ, and FSIQ (Additional file 2). The data for the two patient groups and the non-retarded (IQ = 70) subgroups revealed statistically significant correlations between the complex tasks and VIQ, PIQ, FSIQ, and VMI, i.e. for the Symbol Search and Coding (focused attention) and for Digit Span and Arithmetic (encoding). Note that also for the WISC subtests Coding and Digit Span, which are not included in the calculation of VIQ, PIQ, or FSIQ, significant correlations were found, as was the case for the VMI. The Bourdon-Vos concentration test revealed no correlations with any of the IQ measures. For the complete groups, the Stroop 3 showed statistically significant correlations with PIQ, but these correlations were absent in the non-retarded patients. None of the results of the simple computerized tasks correlated with VIQ, PIQ, or FSIQ.

## Discussion

The present study investigated various dimensions of attention in children with spina bifida with myelomeningocele (SBM) and associated cerebral malformations and in a matched peer group without cerebral malformations (SB). To separate attention from other, interfering functions we compared their performance on traditional attention tasks requiring more complex cognitive and visual-motor skills with their output on comparable but computerized attention tasks that only required a button press in response.

The SBM patients performed significantly less well than their SB counterparts on the traditional attention tasks of the focused attention, encoding and distractibility/impulsivity domain, confirming earlier reports. Because of the complex response requirements required, Fletcher *et al. *[19] had already highlighted the need to separate motor functions from attention by using computer-based tasks. In the computer tasks (ANT), interference from visual-motor and cognitive skills was greatly reduced while the essential aspects of the attention functions were preserved. The resulting data indeed provided new information on the measurement of attention. For the focused attention and encoding domain measured with traditional tasks, highly significant differences between SBM and SB, and large effect sizes were found. A moderately significant difference was found for the distractibility/impulsivity domain. These traditional tasks require rapid visual scanning (symbol search and coding), forward and backward verbal reproduction (digit span), calculation skills (arithmetic), and rapid verbal naming (Stroop 3). In the corresponding ANT tasks, which do not require these acquired cognitive skills, only a few significant differences between children with SBM and SB were found. Thus, the outcomes on the ANT subtests showed only a moderately significant difference between the two patient groups on the focused attention tasks, with medium effect size for the reaction time (RT) measure only. In the other domains no significant differences between SBM and SB on ANT subtests were found, and only a single moderate effect size on sustained attention reaction time. Thus, when the children's visual-motor and acquired cognitive skills were less implicated, performance differences almost disappeared.

The correlational analysis confirmed this result. The performance data on the traditional tasks in the domains of focused attention and encoding correlated moderately to highly with the children's IQ scores and their scores on the visual-motor integration test (VMI). Very small and non-significant correlations with cognitive skills were found for the corresponding computerized attention tests. Together, these results support the hypothesis that attention scores as measured by traditional tasks were confounded by deficits in other visual-motor or cognitive skills. Interestingly, the results on the Bourdon-Vos Test, a paper-and-pencil task, were similar to the results on the computer tasks in that they showed no correlations with the intelligence measures. Also, no difference between children with SBM and SB were found on this task, which in different studies has been shown a valid measure of attention [33,34]. So this test seems to be an adequate measure of sustained attention also for children with SBM.

A similar, but less distinct, result was found with respect to the domain distractibility/impulsivity. Although a significant correlation with intelligence was found for Stroop3, and a significant difference in combination with large effect size between SBM and SB, very low correlations with intelligence and only a small effect size was found for the Stroop interference scores. The latter score is calculated by subtracting speed of naming colours from naming speed in the interference condition, thus correcting for the possibly confounding effect of naming speed.

To control for general cognitive impairment, the data of the non-retarded children (IQ ≥ 70) were analyzed separately. The results provided further support for the interference of visual-motor and acquired cognitive skills in the traditional attention tests: the performance differences between the non-retarded SBM and SB patients were much smaller than the differences observed in the complete patient groups. In the non-retarded patients, statistically significant differences between SBM and SB were only found for the focused attention domain as assessed by the traditional tasks. There were no statistically significant differences between the two non-retarded subgroups on any of the attention domains as assessed by computerized tasks, and effect sizes were small. Together, these results strongly suggest that the measurement of attention is confounded by acquired cognitive and visual-motor skills, especially for children with impairments in these skills.

Summing up, our results confirm earlier claims that most traditional attention tasks also place a high load on visual-motor and acquired cognitive skills. This makes the tasks less valid for the assessment of attention in paediatric populations with cerebral malformations, such as is the case in SBM.

The most robust difference between the children with SBM and those with SB concerned focused attention as measured with complex tasks. First, it should be noted that the children with SB scored within normal limits on the complex focused attention tasks (the normalized score being 10), whereas the children with SBM scored significantly lower. According to Brewer *et al. *[18] and Fletcher *et al. *(1996) impaired focused attention might reflect a deficient posterior attention system. In SBM, neuropathological changes such as hydrocephalus, a reduction in cerebral white matter and overall cortical mantle, and other cerebral anomalies, particularly concern the posterior part of the brain. These are the same neurological structures that are involved in the extensive psychomotor deficiencies commonly observed in these children (see also Loss *et al*, [20]). Thus, based on our and earlier studies, especially in the focused attention domain, the use of tasks that require uncomplicated responses are imperative in order to prevent the attention measures from being confounded by visual-motor deficits.

Conclusions about brain-behaviour relationships are complicated by the complexity of associated neuropathology in SBM. Frequently, associated malformations involve hydrocephalus, Arnold-Chiari II malformation, and corpus callosum dysgenesis. On the basis of the literature on both hydrocephalus (caused by other etiologies than spina bifida) and cerebellar malformations, Vinck *et al. *[28] offered further interpretations about the association between the specific contribution of the different pathological conditions and particular cognitive deficits. Applied to the present results, the poor performance on the more complex traditional attention tasks could be related to cerebellar brain defects influencing motor speed and coordination [37], as well as to the transfer of information across the corpus callosum. Given the associations of the cerebellum and corpus callosum with executive functions, learning and attention, our results could also be interpreted to indicate that attention functions deteriorate in more demanding task situations. Such an interaction between decreasing attention with increasing task-complexity stresses the importance of neuropsychological assessment of functions both separately and combined in diverse contexts.

## Conclusion

In conclusion, both in experimental settings and in clinical practice, attention has proven a difficult concept to assess accurately in patients with SB and SBM. This is due to confounding by and interactions with motor and cognitive demands of most traditional attention tasks, which do not disentangle the effects of visual-motor and verbal deficits in the cognitive profile associated with spina bifida. The current results show how we can separate the role of attention from the effects of complex neuropsychological impairments by reducing the complexity of the task demands. Therefore, attention should be measured by means of computerized reaction time tasks that place little or no demands on visual-motor or acquired cognitive functions.

## Abbreviations

ANT: Amsterdam Neuropsychological Tasks; CT: computed tomography; FSIQ: full-scale intelligence quotient; MRI: magnetic resonance imaging; PIQ: performance intelligence quotient; SB: spina bifida; SBM: spina bifida with myelomeningocele; VIQ: verbal intelligence quotient; WISC-III: Wechsler-Intelligence Scale for Children, 3rd edition Dutch version.

## Competing interests

The authors declare that they have no competing interests.

## Authors' contributions

AV participated in the conception and design of the study, collected the data (administered the neuropsychological tests), performed the statistical analyses and drafted the manuscript.

RM participated in the conception and design of especially the neurological aspects of the study, and helped to draft the manuscript. JR participated in the conception, design and coordination of the study, and helped to draft the manuscript. BM conceived of especially the neuropsychological aspects of the study, participated in the design, coordination and analyses, and helped to draft the manuscript. All authors have read and approved the final version of the manuscript.

## Supplementary Material

Additional file 1 **Table S1.** Medians (interquartiles) per attention domain and task type for the complete SB and SBM groups and the non-retarded subgroups. Click here for file

Additional file 2 **Table S2.** Spearman's rho correlations between VIQ, PIQ, FSIQ, VMI and the attention tasks for the complete SB and SBM groups and the non-retarded subgroups. Click here for file
