# Supplementary material for: Neuropsychological assessment of attention in children with spina bifida
Source: Cerebrospinal Fluid Res. 2009 May 28;6:6. doi: 10.1186/1743-8454-6-6 (PMC2700079; doi:10.1186/1743-8454-6-6)
Supplement: Additional file 1 — Table S1. Medians (interquartiles) per attention domain and task type for the complete SB and SBM groups and the non-retarded subgroups. [file 1743-8454-6-6-S1.doc]

**Table S1.** Medians (interquartiles) per attention domain and task type for the complete SB and SBM groups and the non-retarded subgroups

| **Attention domain** | **Type** | **Subtest** | **SB** | **n** | **SBM** | **n** | **p** | **ES** | **IQ≥70SB** | **n** | **IQ≥70SBM** | **n** | **p** | **ES** |
| --- | --- | --- | --- | --- | --- | --- | --- | --- | --- | --- | --- | --- | --- | --- |
| ***Focused attention*** | Complex | Symbol Search (WISC-III) | 10.5 (4) | 20 | 6.5 (6) | 24 | **<0.001** | 0.20 | 10.5 (3) | 18 | 8.0 (4) | 15 | **<.001** | 0.24 |
|  |  | Coding (WISC-III) | 8.5 (4) | 20 | 4.0 (6) | 24 |  | 0.18 | 9.5 (3) | 18 | 6.0 (7) | 15 |  | 0.24 |
|  | Simple | ANT: Focused Attention Error | 3.5 (5) | 20 | 4.0 (6) | 24 | **<0.05** | 0.44 | 4 (5) | 18 | 3 (7) | 15 | n.s. | 0.49 |
|  |  | ANT: Focused Attention RT^+^ | 1074 (588) | 20 | 1406 (616) | 24 |  | 0.30 | 1120 (608) | 18 | 1233 (1021) | 15 |  | 0.40 |
| ***Sustained attention*** | Complex | Bourdon-Vos Row Time | 15.5 (8) | 12 | 21.0 (18) | 14 | n.s. | 0.38 | 15.5 (7) | 10 | 15.0 (8) | 9 | n.s. | 0.43 |
|  |  | Bourdon-Vos SD | 2.0 (1) | 12 | 3.0 (6) | 14 |  | 0.29 | 2 (1) | 10 | 2 (2) | 9 |  | 0.43 |
|  | Simple | ANT: Sustained Attention Error | 14.5 (8) | 12 | 16.0 (31) | 15 | n.s. | 0.41 | 17 (10) | 10 | 15 (16) | 9 | n.s. | 0.43 |
|  |  | ANT: Sustained Attention RT^+^ | 681 (248) | 12 | 830 (235) | 15 |  | 0.30 | 681 (248) | 10 | 735 (266) | 9 |  | 0.40 |
| ***Encoding*** | Complex | Digit Span (WISC-III) | 10.0 (5) | 20 | 7.0 (5) | 25 | **<0.005** | 0.30 | 10.5 (4) | 18 | 8.5 (4) | 16 | n.s. | 0.36 |
|  |  | Arithmetic (WISC-III) | 9.0 (4) | 20 | 6.0 (6) | 25 |  | 0.27 | 9.5 (4) | 18 | 8.5 (5) | 16 |  | 0.38 |
|  | Simple | ANT: Memory Search Error | 2.0 (3) | 20 | 2.0 (3) | 25 | n.s. | 0.46 | 1.5 (3) | 18 | 1.5 (2) | 16 | n.s. | 0.48 |
|  |  | ANT: Memory Search RT^+^ | 932 (599) | 20 | 1059 (343) | 25 |  | 0.38 | 932 (607) | 18 | 1054 (498) | 16 |  | 0.43 |
| ***Distractibility/*** | Complex | Stroop 3^+^ | 142 (65) | 15 | 190 (81) | 23 | **<0.05** | 0.29 | 142 (51) | 13 | 173 (57) | 14 | n.s. | 0.38 |
| ***Impulsivity*** |  | Stroop Interference^+^ | 60 (27) | 15 | 74 (51) | 23 |  | 0.38 | 60 (24) | 13 | 72 (50) | 14 |  | 0.43 |
|  | Simple | ANT: GoNoGo Error | 2.0 (3) | 20 | 2.0 (2) | 27 | n.s. | 0.44 | 2 (4) | 18 | 1.5 (2) | 18 | n.s. | 0.42 |
|  |  | ANT: GoNoGo RT^+^ | 448 (147) | 20 | 493 (169) | 27 |  | 0.40 | 459 (167) | 18 | 475 (212) | 18 |  | 0.47 |

*Notes:*

WISC-III = Wechsler-Intelligence Scale-III; ANT = Amsterdam Neuropsychological Tasks

^+^ Scores are based on speed measures (with high scores reflecting poor performance)

RT = reaction time; SD = standard deviation; SB = spina bifida; SBM = spina bifida with myelomeningocele

ES = effect size based on Mann-Whitney *U* statistics; benchmarks: <0.29 = large ES; <0.36 = medium ES; <0.44 = small ES; <0.50 = no difference [36]
