# Supplementary material for: Neuropsychological assessment of attention in children with spina bifida
Source: Cerebrospinal Fluid Res. 2009 May 28;6:6. doi: 10.1186/1743-8454-6-6 (PMC2700079; doi:10.1186/1743-8454-6-6)
Supplement: Additional file 2 — Table S2. Spearman's rho correlations between VIQ, PIQ, FSIQ, VMI and the attention tasks for the complete SB and SBM groups and the non-retarded subgroups. [file 1743-8454-6-6-S2.doc]

**Table S2.** Spearman’s rho correlations between VIQ, PIQ, FSIQ, VMI and the attention tasks for the complete SB and SBM groups and the non-retarded subgroups

|  |  |  | **Complete groups** | | | | | **Non-retarded groups** | | | | |
| --- | --- | --- | --- | --- | --- | --- | --- | --- | --- | --- | --- | --- |
| **Task type** | **Attention domain** | **Subtest** | **VIQ** | **PIQ** | **FSIQ** | **VMI** | **n** | **VIQ** | **PIQ** | **FSIQ** | **VMI** | **n** |
| **Complex** | Focused attention | Symbol Search | ***0.62***** | **0.75**** | **0.72**** | **0.64**** | 51 | ***0.38**** | **0.63**** | **0.56**** | **0.45**** | 37 |
|  |  | Coding | ***0.61***** | **0.79**** | **0.75**** | **0.61**** | 51 | 0.29 | **0.58**** | **0.51**** | 0.27 | 37 |
|  | Sustained attention | Bourdon-Vos Row Time | -0.15 | -0.28 | -0.21 | -0.19 | 45 | 0.16 | -0.03 | 0.10 | 0.04 | 34 |
|  |  | Bourdon-Vos SD | -0.09 | -0.27 | -0.17 | -0.22 | 45 | 0.27 | -0.03 | 0.15 | 0.02 | 34 |
|  | Encoding | Digit Span | ***0.66***** | **0.60**** | **0.69**** | **0.58**** | 51 | ***0.46***** | **0.35*** | **0.48**** | 0.32 | 37 |
|  |  | Arithmetic | ***0.84***** | **0.74**** | **0.84**** | **0.70**** | 51 | ***0.71***** | **0.48**** | **0.70**** | **0.45**** | 37 |
|  | Distractibility/ Impulsivity | Stroop 3 | -0.18 | **-0.34*** | -0.30 | -0.13 | 38 | 0.27 | -0.06 | 0.09 | 0.21 | 27 |
|  |  | Stroop Interference | -0.01 | -0.12 | -0.08 | 0.01 | 38 | 0.38 | 0.01 | 0.18 | 0.15 | 27 |
| **Simple** | Focused attention | Focused Attention Error | 0.06 | -0.07 | -0.01 | 0.06 | 45 | 0.14 | 0.001 | 0.07 | 0.18 | 34 |
|  |  | Focused Attention RT | 0.04 | -0.23 | -0.09 | -0.16 | 44 | 0.32 | -0.09 | 0.14 | -0.03 | 33 |
|  | Sustained attention | Sustained Attention Error | -0.03 | -0.32 | -0.24 | -0.15 | 27 | 0.40 | -0.05 | 0.20 | 0.30 | 19 |
|  |  | Sustained Attention RT | -0.31 | -0.34 | -0.36 | -0.22 | 27 | -0.22 | -0.14 | -0.19 | 0.04 | 19 |
|  | Encoding | Memory Search Error | 0.02 | -0.17 | -0.09 | -0.004 | 45 | 0.28 | 0.001 | 0.13 | 0.24 | 34 |
|  |  | Memory Search RT | 0.03 | -0.10 | -0.01 | -0.06 | 45 | 0.20 | -0.05 | 0.12 | 0.002 | 34 |
|  | Distractibility/ Impulsivity | GoNoGo Error | 0.09 | 0.04 | 0.04 | 0.17 | 47 | 0.04 | -0.05 | -0.04 | 0.14 | 36 |
|  |  | GoNoGo RT | 0.01 | -0.09 | -0.05 | 0.03 | 47 | 0.14 | -0.01 | 0.08 | 0.14 | 36 |

*Notes:*

* Correlation is statistically significant at the 0.05 level (2-tailed)

** Correlation is statistically significant at the 0.01 level (2-tailed)

VIQ = verbal intelligence quotient; PIQ = performance intelligence quotient; FSIQ = total intelligence quotient; VMI = Visual-Motor Integration score ; RT = reaction time ; SD = standard deviation

Note that Coding and Arithmetic are subtests of the Wisc-III intelligence test, and included in the calculation of FSIQ, and PIQ and VIQ respectively.
